# Supplementary material for: Exogenous Sorbitol Application Confers Drought Tolerance to Maize Seedlings through Up-Regulating Antioxidant System and Endogenous Sorbitol Biosynthesis
Source: Plants (Basel). 2023 Jun 26;12(13):2456. doi: 10.3390/plants12132456 (PMC10346475; doi:10.3390/plants12132456)
Supplement: Supplementary file 1 [file plants-12-02456-s001.zip › plants-2418757-supplementary.pdf]

plants MDPI

Table S1. Primers used for qRT-PCR analysis.

Gene	Primer sequence	
	**Forward (5′-3′)**	**Reverse (5′-3′)**
ZmAR1	ATTGTGCCAGAGTTTACG	TCATTCAGGCTGTCATTT
ZmAR2	AGAGGTCGGGTTTGGTCTCA	TCCTTCAGTGTGCCGTTCAG
ZmAR3	GAGAAAGAGGTCGGTAGAGG	AAGGGCCAGTGGATAAGG
ZmAR4	GATTGTGCCAGAGTTTACG	TCATTCAGGCTGTCATTT
ZmAR5	CTCGGGAGGAGGTGTTCGT	TGCTTGGTCCTGAAGTTGCT
ZmAR6	CTCCCGTGGTCAATCAGGTT	TCTTCACGATCAGGCAGTCG
ZmAR7	CAAGGTGCTCTGACGGGAAA	GGTTGAAGTCCTCCCGTAGC
ZmAR8	AAGAACCTAGCACACGACCC	GACGGACCACCTTGGACTTG
ZmAR9	ACAAACTGGACAAGACCCCG	CGGCAGGTACAACATGCAAA
ZmActin	GTCCATGAGGCCACGTACAA	CCGGACCAGTTTCGTCATA
